# Twenty‐One Years and Still Going Strong: A Qualitative Study Exploring the Contribution of Young Adult, Adolescent, and Stakeholder Involvement to the Resilience of a Type 1 Diabetes Transition Program

**DOI:** 10.1111/hex.70419

**Published:** 2025-08-29

**Authors:** Ann Carrigan, Veslemøy Guise, D. Jane Holmes‐Walker, Kaye Farrell, Ann M. Maguire, Siri Wiig, Nehal Singh, Shalini Wijekulasuriya, Putu Novi Arfirsta Dharmayani, Zach Simone, Elizabeth Davis, Timothy W. Jones, Tony Huynh, David E. Bloom, Jeffrey Braithwaite, Yvonne Zurynski

**Affiliations:** ^1^ Centre for Healthcare Resilience and Implementation Science, Australian Institute of Health Innovation Macquarie University Sydney New South Wales Australia; ^2^ SHARE—Centre for Resilience in Healthcare, Faculty of Health Sciences University of Stavanger Stavanger Norway; ^3^ Westmead Hospital Sydney New South Wales Australia; ^4^ Sydney University Medical School Sydney New South Wales Australia; ^5^ The Children's Hospital at Westmead Sydney New South Wales Australia; ^6^ Partnership Centre for Health System Sustainability, Australian Institute of Health Innovation Macquarie University Sydney New South Wales Australia; ^7^ Children's Diabetes Centre, Telethon Kids Institute The University of Western Australia Perth Western Australia Australia; ^8^ Department of Endocrinology and Diabetes Perth Children's Hospital Perth Western Australia Australia; ^9^ Division of Paediatrics, School of Medicine The University of Western Australia Perth Western Australia Australia; ^10^ Department of Endocrinology & Diabetes Queensland Children's Hospital South Brisbane Queensland Australia; ^11^ Children's Health Research Centre, Faculty of Medicine The University of Queensland South Brisbane Queensland Australia; ^12^ Department of Global Health and Population Harvard T.H. Chan School of Public Health Boston Massachusetts USA

**Keywords:** resilience in healthcare, transition, transitional care, type 1 diabetes, young adults

## Abstract

**Background:**

Adolescence is a period of rapid transformation when meeting targets for optimal diabetes care is often challenging due to competing life demands. For more than two decades a diabetes transition clinic in Sydney, Australia, has sustained positive outcomes and demonstrated aspects of resilience in the care of individuals living with type 1 diabetes (T1D) who have transitioned from paediatric to adult care. Many studies have focused on resilience in acute care setting showever, studies that examine the factors that support resilience in settings that care for individuals with long‐term, chronic conditions such as T1D are lacking.

**Objective:**

This study aims to examine the contributions of relationships among stakeholders, including adolescents and young adults living with T1D, their carers and healthcare providers, to the resilience of a T1D transition clinic.

**Methods:**

Clinic observations at a paediatric clinic (transition preparation) and a T1D transition clinic located in an adult hospital, and interviews with eight providers comprising endocrinologists, diabetes educators, nurses and an administrator, and 17 adolescents/young adults (aged 16–25 years) living with T1D were conducted. Inductive and deductive analyses were performed using a framework of seven principles for Patient and Stakeholder Involvement in Resilience in Healthcare.

**Results:**

There was evidence for all seven principles though three were strongly expressed in the data: (1) Individual factors, (2) Influence of leadership and (3) Relationships among young adults, carers, and healthcare providers. Overall, key contributions to the clinic's resilience included a stable and cohesive team, continuity of staff, strong leadership sustained over 20 years, presence of a clinic coordinator, open channels of communication, and a positive, supportive culture among clinic stakeholders. There was also evidence of situational resilience and established processes that were adapted to suit an individuals' needs such as transition timing, mode of service (e.g., telehealth) and appointment time (e.g., after hours).

**Conclusion:**

Resilience in transitional care settings such as those that support young adults living with T1D are contingent on person‐centred care, relational leadership, and strong and supportive relationships. Similar transitional care clinics could seek to test this model in different contexts to assess the transferability of the findings described here.

**Abbreviations**
AYA: Adolescent and Young Adult clinic
CGM: continuous glucose monitor
DKA: diabetes ketoacidosis
HbA1c: glycated haemoglobin
ISPAD: International Society for Pediatric and Adolescent Diabetes
MDT: multidisciplinary team
PSI: Patient and Stakeholder Involvement
RiH: resilience in healthcare
T1D: type 1 diabetes
YAs: young adults

## Patient and Public Contribution

Providers working in a diabetes clinical environment and a young person with lived experience of T1D provided feedback on the draft interview guide and participant information letter for this study.

## 1 Introduction

Type 1 diabetes (T1D) is a chronic and incurable autoimmune disease. In 2021, there were a reported 8.4 million individuals living with T1D worldwide and 18% were aged < 20 years [1]. Although most individuals understand the importance of glycaemic control in achieving long‐term benefits, registries show that less than 20% achieve recommended glycaemic targets [1, 2]. This can lead to a reduced life expectancy of 12–16 years compared with non‐T1D persons, and other chronic diseases such as cardiovascular disease [3]. T1D is predominantly diagnosed in childhood [1] but as the condition is lifelong, young adults (YAs) need to transition to adult services. However, the transition from paediatric to adult care can be difficult to navigate for YAs largely attributed to competing life stressors related to tertiary education, employment, relationships, and social demands [3]. Consequently, treatment adherence rates for YAs fall significantly, before and during transition which places them at high‐risk for T1D‐related complications such as diabetes ketoacidosis (DKA) [4].

The International Society for Pediatric and Adolescent Diabetes (ISPAD) guidelines for diabetes in adolescence recommend that services should acknowledge that this developmental stage requires a targeted approach [4]. Some observers suggest that failures in the translation of successful interventions into practice and in the implementation of comprehensive and sustainable transition models of care may contribute to these findings [5, 6]. A recent systematic review by Zurynski et al. [7] on T1D transition models of care found mixed results and gaps in the reporting of implementation outcomes such as model adoption and acceptability were identified. Transition clinics that offer a model of care that provides specific components to support YAs during transition are increasingly utilised, with evidence for improved clinical and psychosocial outcomes [8, 9].

For the past 21 years, a YA T1D transition care clinic located in a tertiary adult referral hospital in Sydney, Australia, has consistently achieved positive clinical outcomes for young people aged 16–25 years [9, 10]. Engagement with the transition care programme, resulted in improved diabetes control with reductions in suboptimal glycaemic levels as measured by glycated haemoglobin (HbA1c), reduced rates of DKA, and reduced unplanned acute hospital admission rates up to 30 months post transition [9, 10]. The adult hospital where the YA T1D clinic is located, is adjacent to a paediatric hospital. The outpatient service is the main source of referrals of YA living with T1D and transitioning to adult care. The transition care programme is delivered via an outpatient clinic by a multidisciplinary team (MDT) led by a senior endocrinologist and a transition coordinator who is also a senior nurse and diabetes educator. As diabetes care is challenging for some YAs, the clinic offers support services such as mobile phone text messaging and/or email appointment reminders, proactive rebooking of missed appointments and after‐hours support for sick day management [10]. It has been suggested that the service model's success is attributed to appropriate support for YAs from a skilled MDT who provides care that is age‐appropriate, flexible and person‐centred, to fit with individuals' needs, preferences, and capacities [9]. As the transition clinic has reported and sustained positive outcomes for YAs living with T1D, this provides a unique opportunity to study and understand the specific components and factors that have contributed to its resilience and sustainability over 21 years.

In 2018, a Resilience in Healthcare (RiH) research programme was established with the overarching goal of identifying the underlying processes involved in the provision of quality healthcare [11]. Wiig and colleagues define resilience in healthcare as ‘the capacity to adapt to challenges and changes at different system levels, to maintain high‐quality care’ [12]. A complex and adaptive system such as healthcare typically comprises multiple interconnected stakeholders who act in predictable and unpredictable ways [13]. During times of uncertainty and when day‐to‐day challenges arise, quality and safety are often maintained by the development of adaptive strategies such as work arounds to anticipate and respond to changing conditions to maintain a functioning system [12, 14].

Understanding the factors that contribute to resilience and sustainability in a T1D transition model of care requires consideration of the perspectives, needs, involvement and contributions of YAs and carers, healthcare providers (doctors, nurses, allied health professionals), and other healthcare system stakeholders (e.g., health managers, regulatory bodies) [15, 16, 17]. Crucially, to understand the resilience and sustainability of models of care, it is important to understand the interconnected relationships among these multiple stakeholders, alongside the barriers and facilitators of programme sustainability [11, 18]. In an inpatient adult T1D setting, specialist healthcare providers were found to be the linchpin for resilient healthcare by bridging gaps, adopting a proactive approach to problem solving, and offering patient support and patient and staff education [19]. Research from non‐diabetes domains such as cancer care has demonstrated how carers play a key role in resilient performance due to their role in information exchange, handover situations, medication administration, nutrition, and mental health support [20]. This supports the notion that relationships among healthcare providers, carers, and individuals contribute to wellbeing and positive health outcomes for YAs.

Guise et al. [21] undertook a stakeholder analysis using data from empirical projects conducted in a variety of care settings to investigate how patients and other healthcare system stakeholders contribute to RiH. Their analysis showed that patients and carers are key stakeholders in the enactment of resilient healthcare services, alongside healthcare professionals and managers [18]. Furthermore, the study found that relationships among stakeholders were crucial for enacting resilience, particularly close ties between individuals and carers, as well as collaborative relationships between individuals, carers and healthcare professionals. From this study, they developed a conceptual framework comprised of seven key principles for Patient and Stakeholder Involvement (PSI) in RiH [15, 16, 17] across different settings that encompass individual, workplace, and organisational elements. Table 1 provides an overview of the main themes and fundamental content of the principles in the framework.

**Table 1:** Deductive analytic framework applied to the observations and interviews based on the seven principles for Patient and Stakeholder Involvement in Resilience in Healthcare [15, 16, 17].

Principle No.	Main theme of principle	Fundamentals of principle
Principle 1	Attention to individual factors	Who is the young person—characteristics and experiences?
		What is their health condition and its complexity?
		Where on the journey are they?
Principle 2	Consider capacity of family and carer involvement	Are family carers involved, or not?
		What factors influence capacity for family involvement?
		Is capacity for family involvement likely to change over time?
Principle 3	Consider healthcare team and staff factors	What competencies and experiences do they have?
		What is the degree of team support?
		What are the circumstances that govern teamwork?
Principle 4	Consider influence of leaders	How do leaders facilitate staff learning and development?
		How aware are leaders of staff workload and needs?
		How do leaders facilitate collegial cultures and relationships?
Principle 5	Assess relationships	Which stakeholder relationships are important?
		What is the degree of contact and collaboration between stakeholders?
		How are relationships between stakeholders valued and facilitated?
Principle 6	Assess the care setting	How does the hospital setting influence adaptations?
		How does the transitional care setting influence adaptations?
Principle 7	Evaluate governance structures	Which laws, policies and guidelines provide context for practice?
		Which laws, policies and guidelines require short‐or long‐term adaptations?

Much of the RiH research has been set in acute care settings such as emergency departments [22]. What is lacking in the literature are studies from other care settings, including long‐term, transitional, primary care, or outpatient settings, as well as research on the contributions of patients and other stakeholders to resilient healthcare. This study aimed to examine the relationships among key stakeholders including adolescents and YAs living with T1D, their carers and health care providers, and their contributions to the resilience of a YA T1D clinic by testing the application of the principles for PSI in RiH framework [17] in an Australian outpatient setting offering paediatric to adulthood transitional care.

## 2 Methods

### 2.1 Setting

The study was conducted at two independent sites in Sydney, Australia: (1) An adult hospital where the transition clinic is located, and (2) a paediatric clinic at a children's hospital, where YAs are prepared for transition. During the period of data collection, the YA T1D clinic offered two afternoon clinics per week (1 p.m.–5 p.m. and 2 p.m.–730 p.m.). The observations took place just after the transition clinic had moved from their usual clinic space to premises within a purpose‐built Adolescent and Young Adult clinic (AYA) space where a skilled MDT provided a ‘one‐stop‐shop’ for young persons living with chronic conditions who are seen four times per year [23]. The YA T1D MDT comprised a paediatric endocrinologist (in the paediatric clinic), a YA endocrinologist, a dietician, specialist nurses, diabetes educators, and administrators. The AYA also provided a social worker and psychologist whom the T1D MDT could access. The transition clinic is supported by a clinic coordinator who is a diabetes educator and specialist nurse who has worked in the clinic for over 20 years.

### 2.2 Study Design

The study was co‐designed with healthcare providers, researchers and a YA living with T1D, ensuring that the research was relevant and meaningful [24]. A qualitative study was conducted using clinic‐based observations and semi‐structured interviews across four stages:

1. Clinic‐based observations.
2. Qualitative interviews with health care providers and teams who provide care to YAs living with T1D.
3. Qualitative interviews with YAs aged 16–25 years, living with T1D who attended the YA clinic at the adult hospital.
4. Qualitative interviews with adolescents (16–18 years) living with T1D and their carers who were being prepared for transition at the paediatric hospital.

### 2.3 Recruitment

Purposive sampling was used to recruit healthcare workers, YAs, and their carers through the transition clinic contacts, social media and email advertisements, and by approaching potential participants in the clinics. Researchers were positioned in the clinic to discuss the research, provide potential participants with an information and consent form to read, and the opportunity to ask questions about the study before consent.

### 2.4 Ethical Considerations

Ethical approval for the study was granted by the Macquarie University Human Ethics Committee (2022/11544) and The Sydney Children's Hospitals Network Human Research Ethics Committee (2023/ETH00404). Governance approval was granted by Westmead Hospital (2023/PID00455, STE00965) and The Children's Hospital at Westmead (STE00964).

### 2.5 Data Collection

#### 2.5.1 Observations

**Figure 1:**
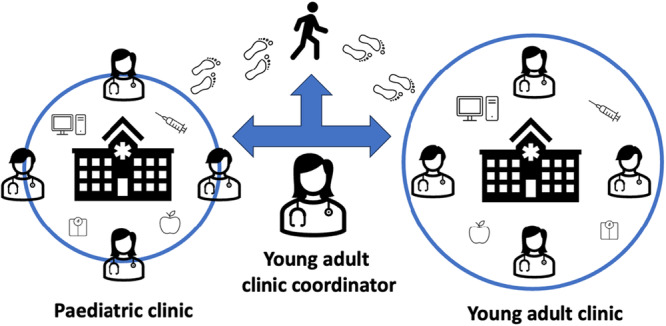
A typical journey of the young adults with T1D from paediatric to adult care, supported by the young adult clinic coordinator.

Three researchers undertook the observations after first participating in two training sessions with the broader research team to discuss what to document. A common understanding of key concepts among observers was achieved through discussions with senior members of the team, before shadowing healthcare workers during clinic visits. Researchers attended the YA T1D clinic for 5 × 5‐h shifts and the paediatric hospital paediatric clinic for 3 × 4‐h shifts in June‐July 2023. Information about clinical interactions including team relations, the characteristics of the key stakeholders including healthcare providers, carers, YAs living with T1D, and organisation were reported. The researchers observed workflows and patient journeys, and reported who was involved in the clinic interactions. Fieldnotes were written into a file on a tablet during the clinic enabling a detailed examination of work‐as‐done. Only general information about workflows and participant perspectives were collected, not individual medical information. At the paediatric hospital, adolescents and YAs were accompanied by a parent, and at the YA clinic around half attended without a parent. The observations revealed interconnected and collaborative MDTs between and within both clinics. The adolescent and YA's journey through the paediatric hospital to the YA clinic was supported by the YA clinic coordinator who facilitated continuity of care (Figure 1).

#### 2.5.2 Qualitative Interviews

Qualitative interviews with consenting providers, adolescents and YAs from each site were undertaken by trained qualitative researchers. The interview guides were pilot tested with two T1D clinicians, researchers, and a YA living with T1D (see File S1). Questions explored what was working well or not so well in the clinic, in addition to examples of practice adaptations in response to challenges and variations in the everyday work setting to maintain the quality and safety of person‐centred care. These could include small adaptations and adjustments done here and now (situated resilience), structural changes that required more resources (structural resilience), and larger changes over time that have been important to adapt the services to the YA with T1D, and how the system is set up around them (systemic resilience) [25]. We also asked about the goal of adaptations, and triggers for, or reasons behind, the adaptations being made. Interviews were audio recorded and transcribed verbatim via Microsoft Teams and lasted ~30 min.

### 2.6 Data Analysis

Fieldnotes were deidentified at the time of recording and aggregated for analysis. All interview audio recordings were transcribed and deidentified, after checking by the research team against the audio recording. Data from the observations and interviews were merged for the analysis. Transcripts were imported into NVivo software [26] for data management and analysis. The transcripts were analysed deductively by authors and qualitative experts using the PSI in RiH framework (Table 1). The coding process involved familiarisation of the themes and fundamentals within each principle and the development of a codebook where the researchers allocated data to the pre‐determined themes [27]. We also undertook an inductive analysis in parallel, to identify data‐driven patterns using an open coding process [28]. Codes included the processes and procedures observed and reported such as YA flow through the clinics and communication mode. Provider, adolescent and YA data were merged for the analysis and the data were synthesised to create a narrative summary. The team worked together to ensure the process was rigorous, discussed major and minor themes and their concomitant categories, and arrived at consensus opinion if any variance in agreement occurred.

## 3 Results

### 3.1 Participants

Seven providers (two from the paediatric hospital, five from the YA T1D clinic), and 12 YAs (one from the paediatric hospital), consented to observation. Eight providers (five from the paediatric hospital, three from the YA T1D clinic) participated in the interviews including endocrinologists (*n* = 2), diabetes educators (*n* = 3) clinical nurse consultants (*n* = 2), [2] and one clinical support officer (administration).

Seventeen participants aged 16–25 years, living with T1D participated in the interviews and included: one being prepared for transition, 15 actively transitioning, and one completing transition. One was still in paediatric care, another was attending the YA clinic for the first time, and one included a parent of an adolescent with special needs. Ages of the YAs ranged from 16 to 25 years; ≤ 18 years (*n* = 5), 19–20 years (*n* = 3), and 21–25 years (*n* = 10), with age of T1D diagnosis ranging from 6 months to 20 years and time since diagnosis ranging from 1 to 21 years (note: the YA diagnosed at age 20 attended the YA clinic only and did not transition from paediatric care). Fourteen participants lived in Northwestern or Western Sydney, one was from Southern Sydney, one from the inner city ( ~ 30 km away), and one from the Central Coast, New South Wales ( ~ 80 km away).

### 3.2 Observations and Interviews

The data aligned with the seven principles for PSI in RiH [16, 17] (Table 2). However, attention to individual factors (Principle 1), the influence of leaders (Principle 4), and relationships between young YAs/carers and healthcare providers (Principle 5) were dominant, and are discussed in detail below.

**Table 2:** Summary of data with quotes supporting the seven principles for Patient and Stakeholder Involvement in Resilience in Healthcare [16, 17].

Principle	Description	Illustrative quotes
1 Attention to individual factors	Transition timing was assessed based on readiness and where the YA was on their T1D journey. Adaptations were made based on their individual needs and preferences.	*I start to think of transition at around the age of 15 and then depending on the maturity of the young person and whether or not they've got other comorbidities, I will bring up transition at a time that I think is appropriate (Paed Clinic, Provider 1)*
2 Consider capacity for family involvement	Parents are involved in the paediatric setting when the transition plan is discussed. During consultations in the clinic, the YAs were mostly independent, however, the parents became involved when required.	*I was forced to take over control myself in the last, I guess, two or three years, because I didn't want my mum hovering, I guess, which is obviously hard for parents of kids with diabetes trying to let go, you know, which is concluding the transition. But yeah, I'm completely independently looking after myself now (YA clinic, YA1)*
3 Consider healthcare team and staff factors	Both sites were staffed with a multidisciplinary team with T1D transition knowledge and skills who ensure they are familiar with each YA and that the care is YA focused.	*The continuity of staff in that there's been one doctor there throughout that time, so that's allowed messaging and had one nurse as well. But the messaging is consistent across the team (YA clinic, Provider 2)*
4 Consider influence of leadership	Service succession plans in place.	*The manager asks me to rotate, train and upskill the staff from the other part of the service in all the technology so that they become more effective in their general role (YA clinic, Provider 1)*
	Evidence of strong, engaged leaders.	
	Holistic organisational leadership focus.	
7 Assess relationships	Evidence of positive relationships among YAs, carers and providers.	*I just find it's almost like a second family. I've been coming here for that long. Yeah, you know, they are always so kind and just compassionate towards if you're going through anything like certain situations, they'll understand, you know, and even with the doctors as well, compassionate, they care. (YA clinic, YA5)*
8 Assess the care setting	Supportive and empowering, accessible, transparent, visible, decisive, no apparent hierarchy.	*I usually just remind them I'm going to see you up until this time and you've probably got three or four more appointments with me. And I ask them ‘are you still happy with that?’, which generally they are. And then once it gets to about nine months before the transition time, we talk about it in more detail. I would then provide a referral letter to the young adult clinic (Paed clinic, Provider 1)*
	YAs are prepared for transition in a T1D outpatient clinic located in a paediatric hospital and then they move to the T1D YA clinic located in an adult hospital where they can be cared for up to age 25 years.	
1 Evaluate governance structures	Process and procedures in place for clinical handovers when staff leave and are replaced by new staff members.	*We've done a lot of continuous quality audit. I think that's been beneficial to inform us of how we're doing, providing services and looking at particular outcomes like attendance, lost to follow up, not just the typical, glycaemic control measures (YA clinic, Provider 2)*
	Adherence to the ISPAD clinical guidelines for caring for YAs living with T1D.	

Abbreviations: ISPAD, International Society for Pediatric and Adolescent Diabetes; T1D, type 1 diabetes; YA, young adult.

### 3.3 Principle 1: Attention to Individual Factors

YAs in this study were at different stages of their T1D journeys, where the time since diagnosis varied between one and 21 years, therefore, there was a range of experience of self‐managing their T1D. Clinic MDTs showed adaptability in accommodating this diversity by flexibly adjusting transition timing based on readiness, psychosocial comorbidities and life events (e.g., major school exams). Providers tailored care accordingly and an information pack was given at transition, and the YAs could remain in paediatric care post age 18 years if needed:

Every resource… it's all to assist the patient's experience, to have that really good experience of support…

(Paed clinic, Provider 2)

In the YA T1D clinic, individualised communication was key. The YAs could text a T1D nurse educator directly for advice and appointment times were flexible. For example, times offered outside of regular working hours to adapt to changing work schedules quickly and easily:

Just being able to send a message at any time to organise appointments, to check my blood sugar levels or graphs for adjustments to carb sensitivity or basal rates or anything like that, just actively quick, convenient.

(YA clinic, YA14)

There was also evidence for adapting the mode of service delivery to YAs' needs and preferences, to ensure a safe, non‐judgemental environment. This supported those who may not be meeting their glycaemic targets, and who subsequently felt reluctant to attend the clinic:

They really make sure that I was looking after myself. Even if, you know, there was weeks where I was like, I don't really want to go because this week I've kind of fallen off the wagon and I didn't really take care of my sugar levels, they (providers) would be like, “Okay, but we're doing it on telehealth, if you're not coming in, we're going to do it on telehealth."

(YA clinic, YP7)

Providers respected YA's choices regarding technology such as continuous glucose monitors (CGM). Some were concerned about their use due to device visibility, discomfort or workplace concerns. Providers explained the benefits of the technology and encouraged them to have a trial but did not place any pressure to do so:

The clinic coordinator has always been asking me about the Libre (CGM), but I've always been a bit anxious, but she kind of, um, she explained how it will help and how much easier will be for me, which eventually I just took the option to do the easier.

(YA clinic, YA10)

Providers avoided judgement when HbA1c and CGM data failed to meet targets. There was acknowledgement that the YAs are doing the best they can and that there are a lot of competing interests at this stage of their lives. Adaptations were also noted when T1D devices (CGMs and insulin pumps) were recommended based on a YA's financial circumstances. Providers noted that access to insulin pumps can be problematic due to the current funding model in Australia where people aged over 18 years can only access insulin pumps via their private health insurance. Providers offered a pump to all YAs if they requested one and were eligible either for supported funding or funding through family private health insurance cover.

### 3.4 Principle 4: The Influence of Leaders on T1D Transitional Models of Care

Leadership played a pivotal role in promoting resilient, high‐quality T1D care across both clinics. Leaders were involved in staff training and capacity building to ensure that the clinical care and culture remains consistent and of a high standard as there is a high degree of workload demands. They also facilitated quality monitoring such as following up with a YA when they did not attend the clinic to ensure that glycaemic levels were in the recommended range. Leaders were forward thinking and considered options for the future to ensure the sustainability of the service by maintaining appropriate staffing levels and succession planning. While the YA clinic lacked formal policies about the management of the YA T1D transition clinic at the hospital level, leaders recognised the need to address this gap to enhance structural resilience.

Organisational accountability was evident in the paediatric clinic with respect to transition:

The push by the organisation has made transition everybody's business*.*

(Paed clinic, Provider 4)

Leaders upheld strong values including integrity, respect, and vision, and these values have been maintained by a strong and consistent leadership team. Leaders also reflected on the need for ongoing adaptations to maintain the established cultural tenets and appropriate staffing levels, in an ever‐changing health system context.

### 3.5 Principle 5: Relationships Among Young Adults, Carers and Healthcare Providers

Strong relationships were observed among all stakeholders. Clear communication between the paediatric and YA T1D clinics facilitated smooth transitions, especially for late T1D diagnoses (e.g., 15–17 years). The transition coordinator was central in managing appointments and supporting YAs and their carers. Decisions were made collaboratively with consultation between the clinics, while taking the young person's and their family's capacity and preferences into consideration.

YAs described providers as mentors rather than authority figures who adopted a proactive approach to involving them in their own care by inviting them to reflect on their health and circumstances, and giving them agency to make decisions (e.g., obtaining a driver's license, insulin adjustments):

Whatever I wanted was my choice and they'd support me…which has been pretty awesome.

(YA clinic, YA3)

A single, consistent team member acted as a trusted point of contact. The YAs appreciated this ongoing access, including after‐hours communication. In the paediatric clinic, communication among staff and carers was maintained via regular emails and follow‐up phone calls. Transition discussions were gradually introduced to YAs in an informal, supportive manner. The communication style between doctors and YA at both clinics was observed to be person‐focused and supportive, using age‐appropriate language and minimising jargon. YAs felt involved in their care decisions and were observed to freely ask questions. When clarification was needed, they were asked open‐ended questions to build their capacity and confidence for self‐management. Providers engage the YA in decision making by discussing their wellbeing and downloading health data collaboratively. When conversing, the provider allowed space for the young person to answer and positioned the computer screen towards them when discussing their data so they could review data together:

They sit down, give you one‐on‐one and like what you should like work on, how you should improve on, like problems that you're having regularly. They'll see the pattern when you upload your data.

(YA clinic, YA 4)

A YA's level of involvement in their care related to their level of experience living with T1D. Also, the relationship with services changes over time as YAs become increasingly active in their own care and related decision‐making. When a YA was experienced with living with T1D, the providers tended to give them agency and respected that they have knowledge about self‐management. For example, a YA identifying patterns in their CGM data comes from experience and they have learned to make the necessary adjustments. Despite providers disagreeing with their choices at times, there was evidence for encouraging autonomy to make adaptations based on the YA's preference and need. This was corroborated by the YAs who felt accepted and welcome at the clinic despite not meeting glycaemic targets. Because of this acceptance and attitude that ‘life happens’, YAs felt welcomed to return and attend the next appointment, which ensures the continuity of YA centred care.

Over time, YAs become more engaged in their care, as the responsibility shifted from parents and providers to the YA. This helped build confidence and promote autonomy. For example, changes to pump settings were made in collaboration with the YA so that they understood the reasons for the decision, which increased their knowledge and therefore capacity for self‐management of care. This also has safety advantages as the YA acts as a second checker for the provider and verifies the adjusted numbers:

The doctor is really good at showing me how they're changing it as well. So not only… I'm saying what's going on. I'm also being taught what's going on.

(YA clinic, YA11)

Providers balanced supportive encouragement with accountability:

I would say I've been very lucky to have them look after me just because I feel like they show a bit of tough love, you know what I mean? I wouldn't really care about my diabetes as much as I should. But they really made sure I did. I feel like the doctors informed me of like, consequences and you know, like the benefits of actually looking after my blood sugar levels, and I saw a massive difference in my health in general. When I wasn't really looking after my diabetes, they would sit me down and tell me like, “This is your case, this is what could happen, this is how you can manage it, this is what it would look like when it's managed, but at the end of the day, it's in your hands.”

(YA clinic, YA7)

## 4 Discussion

This study examined resilience in healthcare in an outpatient YA T1D transition clinic setting through the lens of a PSI in RiH ramework, to understand how the quality and safety of the clinic have been sustained over a long period. Our analysis found evidence for all seven principles for PSI in RiH [17]. However, three dominant principles emerged as crucial for resilience within the YA T1D clinic: Attention to individual factors (Principle 1), the influence of leaders (Principle 4), and relationships between young YAs/carers and healthcare providers (Principle 5). We found that carer support, the presence of consistent staff including a transition coordinator, open and flexible channels of communication, adaptations to a YA's needs and preferences, and the maintenance of a positive culture for YAs and staff made important contributions to a successful transition. We also found strong links among the principles with evidence for several person and service‐initiated adaptations. Our findings align with the principles and policies of good transition care for YA living with non‐T1D chronic conditions [29, 30], and places the YA at the centre of the transition process to ensure continuity of care.

There has long been an emphasis on the need for flexible, patient‐centred transition models [31], and this is especially important in a T1D context where there is a large amount of variation in a YA's ability to adapt and respond to change [7]. Our findings extend this study by providing granular examples of how such care is operationalised and an understanding of ‘one size does not fit all’. For example, delaying transition for school exams, offering telehealth for disengaged YAs, and texting the clinic coordinator directly. Traditional models often emphasise ‘readiness for transition’ as a fixed milestone, whereas the YA T1D model is fluid, context dependent, and best assessed via ongoing relational interactions, not checklists alone. The adaptations made support YAs during the transition period, has helped increase their confidence in self‐management of T1D, that ultimately improves their health and well‐being.

The findings reflect a highly responsive, relationship‐centred model of care that aligns well with the resilience principles. We have also highlighted how resilience is not only structural (e.g., staffing) but is deeply situated. Although the model of care and key staff have remained unchanged for 21 years in this clinical setting, we observed daily, interpersonal care adaptations that reflect micro‐level responses to individual needs. For example, YAs' carers step in when they cannot contribute themselves; providers invite YAs in as decision‐makers and adapt to meet their needs and preferences; staff adapt to each other's needs and abilities; leaders accommodate their staff and make adaptations according to the needs of the staff and the clinic. These findings support the notion that providers, and in particular service leaders [22], are key to supporting the YA's transition to adult care by facilitating the accessibility and consistency of the transition service.

There was evidence of leadership enablers for adaptive capacity as presented in the leadership framework developed by Fagerdal et al. [32]. Specifically, this framework entails four key leadership activities that underpin adaptive capacity in teams: *Situational understanding of work practice needs*—leaders ensured that work was efficient with clear processes and that the workload of staff was adequate and adjusted depending on staff availability and clinic attendance; *Building competence*—leaders provided adequate training for new staff that included how to support YAs to build capacity for self‐care; *Balancing workload, risk and staff needs*—leaders ensured that experienced team members shared knowledge and skills among the less experienced members and that individual staff needs where supported; *Relational leadership—*leaders were present in the clinic(s) and fostered a culture of involvement, caring and willingness to help others among the staff. However, more research is needed to assess the links between the organisation and leadership of services, and the relationships formed between YAs and care teams, and the implications of this for the resilient delivery of health services and to achieve desired health outcomes for YAs.

Across both clinics there was limited access to social workers, counsellors and psychologists as reported by participants and observed by researchers; this is consistent with previous findings [7]. Providers can manage crisis interventions but preventative and ongoing counselling about diabetes burnout, brought about by the psychological and physical demands of managing diabetes [33] is not offered. In response, providers arrange a brief one‐off session for the YA with a social worker and link them to local psychologists (outside of clinic), who they would see after referral from their primary care provider. However, it was felt that this process is not ideal as the counsellor may not have T1D‐specific knowledge. Additionally, there is an out‐of‐pocket expense involved so will not be an option for some YAs. Access to shared services within the AYA MDT including a social worker and psychologist was available, although not observed in the current study. However, this service may be limited as they are not likely to have the T1D‐specific expertise needed, such as a managing the expectations for independent diabetes management.

The model of care presented here aligns with the key principles for YA transitional care in other chronic health conditions (e.g., asthma) [29]. Specifically, early preparation, empowerment, enabling of self‐care, presence of a coordinator, good communication and shared responsibility and person‐centred care. However, our observations revealed a potential risk in the event of leadership change. Although succession plans are in place, the limited formal documentation of the T1D YA clinic in the broader hospital service plans, and structured institutional support poses a risk to sustainability and may lead to burnout.

## 5 Strengths and Limitations

This study empirically tests and provides support for a RiH framework in an outpatient setting and provides valuable insights for transition care within the T1D context, work that has previously been lacking in the literature [34]. Adopting a co‐design approach involving endocrinologists and a YA living with T1D strengthens this study. However, there are certain limitations that must be noted. This study was conducted in tertiary, urban hospitals which are well resourced compared to healthcare facilities in regional and remote settings. As the focus is on urban T1D settings, the application to other clinical settings may be limited. Further, it was conducted in an English‐speaking, high‐income country [35]. Exploring perspectives from low‐ or middle‐income countries, including participants who are from a non‐English speaking background would broaden the knowledge about PSI in achieving RiH in T1D transition services. Examining these factors in terms of participants' socioeconomic status and education level would be beneficial. Despite these limitations, there are several key findings that could be applied in smaller, low‐income, non‐English speaking settings: Strong leadership, empowering relationships between the YAs and providers to capacity build for self‐care, and the importance of a central co‐ordinator to prevent YAs from falling through the gaps. Additionally, the study was based on a single service within a very specific context including the paediatric services as being next‐door to the adult service, therefore transferability of these results may not hold true in other contexts. Finally, other key stakeholders such as policymakers and hospital administrators were not included in the current study. Future work that considers these stakeholders would broaden the findings, specifically in relation to principle 7 of the PSI RiH framework, ‘Evaluation of governance structures’.

## 6 Implications

This study makes a significant contribution to the literature by empirically testing the PSI in RiH framework to understand the factors involved in resilience and sustainability in a transitional care model for YAs with T1D. It also has practical and educational implications, as we have shown how adolescents, YAs, carers, and providers interact to facilitate and enact the adaptations needed to maintain high‐quality care. Consistent and skilled staff led by a clinic coordinator, open channels of communication, flexibility and a positive culture supported adolescents and YAs to build capacity for self‐management of their T1D. Importantly, this study provides an understanding of the mechanisms underpinning the successful transition from paediatric to adult care. These findings may therefore be of interest in similar work on how young persons with other long‐term or chronic conditions transition from paediatric to adult care, such as cancer, asthma, epilepsy, renal disease, and cerebral palsy.

## 7 Conclusion

This study empirically tested a RiH framework to understand the factors involved in the resilience of a T1D transition clinic. The findings show that successful care requires more than structured pathways. Respect for individual preferences, flexibility, trusting relationships, and strong leadership make important contributions to safety and quality. There was evidence for situated, structural and systemic resilience with interactions to facilitate and enact the adaptations needed to maintain high‐quality care. Consistent and skilled staff led by a clinic coordinator, open channels of communication, flexibility and a positive culture supported adolescents and YAs to build capacity for self‐management of their T1D. Importantly, this study provides an understanding of the mechanisms that may be involved in the successful transition from paediatric to adult care for all chronic health conditions.

## Author Contributions

**Ann Carrigan:** conceptualisation; methodology; formal analysis; validation; project administration; writing – review and editing; writing – original draft; investigation; data curation; resources. **Veslemøy Guise:** validation; writing – review and editing; data curation; formal analysis. **D. Jane Homes‐Walker:** conceptualisation – subject matter expertise; writing – review and editing; validation. **Kaye Farrell:** conceptualisation – subject matter expertise; writing – review and editing; validation. **Ann M. Maguire:** conceptualisation – subject matter expertise; writing – review and editing; validation. **Siri Wiig:** formal analysis; validation; writing – review and editing; **Nehal Singh:** project administration; writing – review and editing. **Shalini Wijekulasuriya:** methodology; validation**:** writing – review and editing. **Putu Novi Arfirsta Dharmayani:** methodology; validation**:** writing – review and editing; validation; **Zach Simone:** conceptualisation – subject matter expertise; writing – review and editing; validation. **Elizabeth Davis:** subject matter expertise; writing – review and editing; validation. **Timothy W. Jones:** subject matter expertise; writing – review and editing; validation. **Tony Huynh:** subject matter expertise; writing – review and editing; validation. **David E. Bloom:** writing – review and editing; validation. **Jeffrey Braithwaite:** writing – review and editing; validation. **Yvonne Zurynski:** conceptualisation; supervision; writing – review and editing; validation. All authors approved the final submitted version of the article.

## Ethics Statement

Ethical approval for the study was granted by the Macquarie University Human Ethics Committee (2022/11544) and The Sydney Children's Hospitals Network Human Research Ethics Committee (2023/ETH00404). Governance approval was granted by Westmead Hospital (2023/PID00455, STE00965) and The Children's Hospital at Westmead (STE00964).

## Consent

All patients provided informed consent to participate.

## Conflicts of Interest

The authors declare no conflicts of interest.

## Permission to Reproduce Material From Other Sources

Not applicable.

## Data Availability

Data may be made available upon a reasonable request to the corresponding author.
